# Comparison of Hematopoietic Stem Cell Transplantation Outcomes Using Matched Sibling Donors, Haploidentical Donors, and Immunosuppressive Therapy for Patients With Acquired Aplastic Anemia

**DOI:** 10.3389/fimmu.2022.837335

**Published:** 2022-02-01

**Authors:** Yuanfeng Zhang, Jiali Huo, Li Liu, Yuyan Shen, Juan Chen, Tingting Zhang, Xin Chen, Aiming Pang, Donglin Yang, Rongli Zhang, Qiaoling Ma, Weihua Zhai, Yi He, Jialin Wei, Erlie Jiang, Mingzhe Han, Yizhou Zheng, Sizhou Feng

**Affiliations:** ^1^State Key Laboratory of Experimental Hematology, National Clinical Research Center for Blood Diseases, Haihe Laboratory of Cell Ecosystem, Institute of Hematology & Blood Diseases Hospital, Chinese Academy of Medical Sciences & Peking Union Medical College, Tianjin, China; ^2^Department of Hematology, The Affiliated Yantai Yuhuangding Hospital of Qingdao University, Yantai, China

**Keywords:** aplastic anemia, transplantation, matched sibling donor, haploidentical donor, immunosuppressive therapy

## Abstract

We retrospectively compared the outcomes of 387 consecutive patients with acquired aplastic anemia (AA) who underwent hematopoietic stem cell transplantation (HSCT) with a fludarabine-based conditioning regimen from matched sibling donors (MSD) (n = 108) or haploidentical donors (HID) (n = 91) and immunosuppressive therapy (IST) (n = 188) from 2014 to 2020 at our hospital. Compared with HID-HSCT, MSD-HSCT had a lower incidence of graft failure (1% *vs.* 7%, *p* = 0.062), grade II–IV acute graft versus host disease (aGvHD) (16% vs. 35%, *p* = 0.001), and mild to severe chronic GvHD (cGvHD) (8% vs. 23%, *p* = 0.007), but an equivalent incidence of grade III–IV aGvHD (8% vs. 12%, *p* = 0.237) and moderate to severe cGvHD (3% vs. 9%, *p* = 0.076). HSCT had superior blood count recovery at 3, 6, and 12 months compared with IST (*p* < 0.001). The estimated 5-year overall survival (OS) of the MSD, HID, and IST groups were 86%, 72%, and 79% (*p* = 0.02), respectively; accordingly, the failure-free survival (FFS) rates were 85%, 68%, and 56%, respectively (*p* < 0.001). For patients aged ≤40 years, the OS rate was still significantly superior for MSD-HSCT receipients compared to HID-HSCT receipients (89% vs. 76%, *p* = 0.024) while the HID-HSCT recipients showed similar OS (76% vs. 78%, *p* = 0.166) but superior FFS (*p* = 0.047) when follow-up was longer than 14.5 months in contrast to IST. In a multivariate analysis, HID-HSCT and a conditioning regimen that included busulfan were adversely related to OS among patients who received allografts. In conclusion, MSD-HSCT was the frontline choice for patients with severe AA aged ≤40 years, while HID-HSCT was as effective as IST for patients without an MSD.

## Introduction

Hematopoietic stem cell transplantation (HSCT) from HLA-matched sibling donors (MSD) is recommended for young and adult patients with severe aplastic anemia (SAA) (1). For those patients without an MSD but with comorbidities or an older age, immunosuppressive therapy (IST) should be considered. However, the efficacy of IST is limited due to treatment non-response, relapse, and clonal evolution (2). Outcomes following alternative donor HSCT, such as haploidentical donor HSCT (HID-HSCT), have improved dramatically over the last decade due to the reduced incidences of graft failure (GF) and graft versus host disease (GvHD). In China, the addition of busulfan (BU) to high-dose cyclophosphamide (CY) (200 mg/kg) combined with rabbit antithymocyte globulin (rATG) 10 mg/kg was an effective conditioning regimen for HID-HSCT and resulted in a similar overall survival (OS) to MSD-HSCT (3) and a superior failure-free survival (FFS) than IST (4, 5). Importantly, for young patients, fertility is a critical issue that may be helped by modifying the conditioning regimen. There is currently no comprehensive comparison of the feasibility and safety of MSD-HSCT, HID-HSCT, and IST for patients with SAA. Further, we used a FAC-conditioning regimen consisting of fludarabine (FLU)+ATG+CY for patients with SAA, with the addition of BU (BFAC) for those with a high risk of GF, such as those with a long disease history, heavy transfusion, and transfusion-dependent non-severe AA (NSAA). We therefore performed a retrospective study to compare the outcomes of MSD-HSCT, HID-HSCT, and IST for patients with AA at our hospital over the same time period.

## Patients and Methods

### Patients

From 2014 and 2020, a total of 387 consecutively patients with acquired AA who received MSD-HSCT (108), HID-HSCT (91), or IST (188) were enrolled into this study. Enrollment criteria included SAA, severe SAA, or transfusion-dependent NSAA as defined by clinical guidelines (6); voluntary use of HSCT or IST; the absence of severe organ dysfunction; and age ≤60 years for HSCT (no age limitation for IST). Excluded were patients with underlying inherited marrow failure disorders such as Fanconi anemia; myelodysplastic syndrome (MDS); pregnant patients; and those with severe organ impairment or an uncontrolled active infection. Patients with paroxysmal nocturnal hemoglobinuria (PNH) clones were also included in this analysis. All written informed consent was attained from the patients or their relatives. This study was approved by the Ethics Committee at our hospital (IIT2021011-EC-1).

### Transplantation and IST Procedures

Transplantation: the FAC conditioning regimen was composed of FLU 150 mg/m^2^ i.v. in divided doses on days -6 to -2, CY 120 or 150 mg/kg i.v. in a divided dose on days -5 to -2, and rATG (Thymoglobulin^®^, Genzyme, Cambridge, MA) 12.5 mg/kg or porcine antilymphocyte globulin (pALG) (Anti-lymphocyte Immunoglobulin^®^, Wuhan Institute of Biological Products Co., Ltd., China) 100 or 125 mg/kg i.v. in divided doses on days -5 to -2. The BFAC conditioning regimen was composed of BU 6.4 mg/kg i.v. in divided doses on days -7 to -6, FLU 150 mg/m^2^ i.v. in divided doses on days -6 to -2, CY 80 to 150 mg/kg i.v. in divided doses on days -5 to -2, and rATG 12.5 mg/kg, pALG 100, or 125 mg/kg i.v. in divided doses on days -5 to -2. We have demonstrated the similar efficacy between rATG and pATG among the MSD-HSCT recipients (7). Since then, selection of ATG was according to the intention of patients and doctors while rATG was mostly used before. Since 2016, the donor-specific anti-HLA antibody (DSA) test was routinely performed during donor selection of HID-HSCT and donors with a negative DSA were chosen. Otherwise, in the absence of an alternative suitable donor, it is recommended that measures against DSAs should be taken (8). Totally, 68 HID-HSCT recipients were identified DSAs and 1 patient was positive. GvHD prophylaxis, infection prevention, and surveillance were in accordance with our previous report (7) and the experience of HID-HSCT performed at other centers in China (4, 5).

IST: The patients in the IST group were treated with rATG at a total dose of 17.5 mg/kg i.v. in divided doses for 9 consecutive days, in line with our previous report (9), or pALG 125 mg/kg i.v. in divided doses for 5 consecutive days. Oral cyclosporine-A (3–5 mg/kg/day) was started 2 weeks after the last day of the rATG/pALG treatment and was administered for at least 2 years (with dose adjustments to achieve a whole blood trough level of 100–200 ng/ml for adults and 100–150 ng/ml for children).

### Definitions

Blood count after therapy was classified as complete response (CR), partial response (PR), and no response (6), and anti-infection response was classified as complete remission, partial remission, stable disease (SD), and progressive disease (10). Days of neutrophil and platelet engraftment (5), acute GvHD (aGvHD) (11), chronic GvHD (cGvHD) (12), and GF (13) were defined according to previously reported criteria. Therapy-related mortality (TRM) was defined as death without relapse. Treatment failures from IST included death, non-response at 6 months and beyond, disease progression requiring intervention, relapse, and clonal evolution (14). Treatment failures after HSCT were defined as death, and primary or secondary GF, whichever came first. FFS was defined as survival without treatment failure. GvHD-free, failure-free survival (GFFS) was defined as survival without grade III–IV aGvHD, moderate to severe cGvHD, or treatment failure (15). OS was defined as the time from treatment start to death or last follow-up.

### Statistical Analysis

The primary objective of this study was to compare the OS of AA patients who received different procedures. Other major outcomes included infection, engraftment, aGvHD, cGvHD, TRM, GFFS, and FFS.

All patients involved had an outpatient department or telephone follow-up. The final date of follow-up was April 30, 2021. Continuous and categorical variables were compared using the Mann–Whitney U, chi-square test, or Fisher exact test, respectively. The cumulative incidence of GvHD and TRM was estimated using a competing risk model and compared with the Gray’s test. Death was considered a competing event for GvHD. The probabilities of OS and FFS were estimated using the Kaplan–Meier method and compared between the different groups of patients using the log-rank test. A landmark analysis was performed when the curves crossed (16). Variables with p values ≤0.05 in the univariate analysis were entered into multivariate analyses using a Cox proportional hazards model to identify factors impacting OS, FFS, and GFFS of transplant patients. According to the results of previous studies (17, 18), variables including patient age, interval from diagnosis to transplantation, RBC transfusions before transplantation, graft source, conditioning regimen, and ATG source were used as covariates in propensity score matching. Patients in the MSD groups were matched to those in the HID group using 1:1 nearest neighbor matching with a caliper width of 0.2. Statistical analyses were performed with the R software package (R 4.0.5), GraphPad Prism 5, and SPSS 20.0 statistical software. GraphPad Prism 5 was also used to generate figures. All *p* values were two-sided, and the results were considered statistically significant when *p* < 0.05.

## Results

### Patient Characteristics

The basic characteristics of enrolled patients and their donors are summarized in **Table 1** and **Supplementary Table 1**. The median ages of patients in the MSD, HID, and IST groups were 26 years (range, 4–54 years), 19 years (range, 4–55 years), and 28 years (range, 7–65 years) (*p* < 0.001), respectively. The IST group had a higher proportion of patients who were older than 40 years (26%) than the HSCT groups (*p* < 0.001 in both cases). The median time from diagnosis to therapy start was 5 months (range, 1–248 months) in the HID group, which was significantly longer than in the MSD (3 months, range, 1–205 months) and IST (2 months, range, 0.4–59 months) (*p* < 0.001) groups. There were no differences between three groups in terms of patient gender and the presence of PNH clones. Details of patients and their donors are provided in **Supplementary Table 1**. The MSD group had a lower rate of male donors (47% vs. 65%, *p* = 0.019) but higher rates of infection (complete remission, partial remission, and stable disease) before HSCT (29% vs. 19%, *p* = 0.009), and younger donors (median age, 26 years vs. 36 years, *p* < 0.001) compared with the HID group. There were also significant differences between the MSD and HID groups in terms of ATG source and the number of mononuclear, CD34^+^, CD3^+^, CD4^+^, and CD8^+^ cells in the graft (**Supplementary Table 1**).

**Table 1:** Characteristics and outcomes of patients with acquired aplastic anemia.

Variables	MSD group (108)	HID group (91)	IST group(188)	*p* value
Patient age, years, median (range)	26 (4–54)	19 (4–55)	28 (7–65)	<0.001
Patient age group (years), no. (%)				<0.001
≤20	41 (38)	49 (54)	57 (30)	
>20 ≤ 40	54 (50)	34 (37)	83 (44)	
>40	13 (12)	8 (9)	48 (26)	
Patient sex (male), no. (%)	68 (63)	53 (58)	105 (56)	0.489
Diagnosis, no. (%)				<0.001
Severe aplastic anemia	60 (56)	51 (56)	97 (52)	
Very severe aplastic anemia	40 (37)	29 (32)	90 (48)	
Non-severe aplastic anemia	8 (7)	11 (12)	1 (1)	
Presence of PNH clones	19 (18)	18 (20)	36 (19)	0.917
Interval from diagnosis to therapy, moths, median (range)	3 (1–205)	5 (1–248)	2 (0.4–59)	<0.001
28-day death, no. (%)	2 (2)	4 (4)	2 (1)	0.208
60-day death, no. (%)	3 (3)	7 (8)	6 (3)	0.184
3-month complete remission of CBC, no. (%)	32 (35)^a^	25 (32)^a^	9 (5)^a^	<0.001
6-month complete remission of CBC, no. (%)	51 (60)^b^	46 (67)^b^	26 (15)^b^	<0.001
12-month complete remission of CBC, no. (%)	46 (78)^c^	46 (82)^c^	43 (31)^c^	<0.001
MDS/AML transformation, no. (%)	0	0	11 (6)	0.002
Follow-up of alive patients, moths, median (range)	37 (4–87)	25 (4–87)	23 (3–87)	0.011

a 91, 77, and 182 evaluable patients.

b 85, 69, and 173 evaluable patients.

c 59, 56, and 140 evaluable patients.

MSD, matched sibling donor; HID, haploidentical donor; IST, immunosuppressive therapy; no., number of patients; PNH, paroxysmal nocturnal hemoglobinuria; CBC, complete blood count; MDS, myelodysplastic syndrome; AML, acute myelocytic leukemia.

### Transplantation Outcomes

Twenty-eight days post-HSCT, 106 of 108 and 87 of 91 patients survived in the MSD and HID groups, respectively. All surviving patients achieved neutrophil engraftment, while patients in the MSD group had a higher rate of 28-day platelet engraftment (94% vs. 76%, *p* < 0.001). The rates of primary graft failure were not significantly different (0 vs. 2%, *p* = 0.202). The median days until neutrophil and platelet engraftment were 12 days (range, 8–19 days) and 13 days (range, 7–37 days) in the MSD group compared with 12 days (range, 10–23 days) (*p* = 0.039) and 14 days (range, 8–95 days) (*p* = 0.023) in the HID group, respectively. MSD patients had a lower incidence of GF compared with HID patients (1% vs. 7%, *p* = 0.062), and one HID-HSCT recipient was diagnosed as primary GF with a median fluorescent intensity of DSA 3735 which was previously reported in another paper (8).

**Figure 1:**
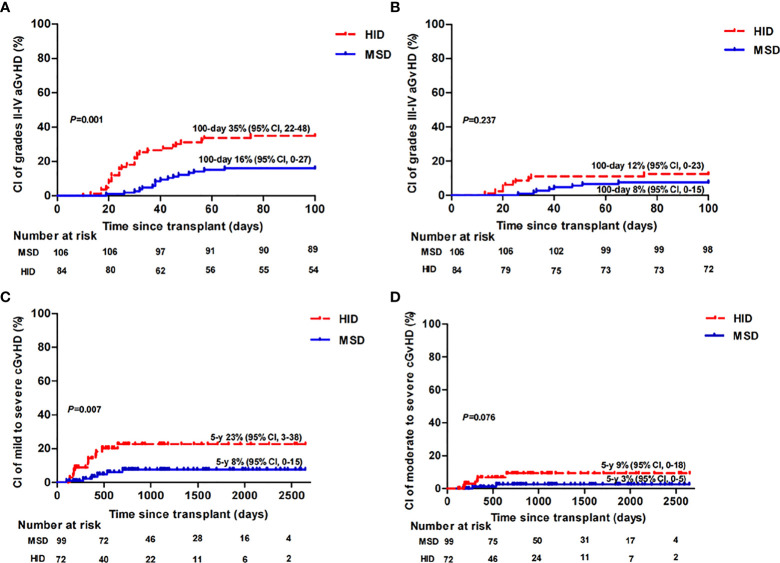
Cumulative incidence (CI) of grade II–IV aGvHD **(A)**, grade III–IV aGvHD **(B)**, mild to severe cGvHD **(C)**, and moderate to severe cGvHD **(D)** in MSD-HSCT and HID-HSCT patients.

Only patients who survived beyond 28 and 100 days after transplantation were evaluated for aGvHD (1 patient in the HID group who experienced grade III aGvHD on day 17 and died on day 26 was included) and cGvHD, respectively. aGvHD occurred in 25 of 106 patients (24%) in the MSD group vs. 59 of 88 patients (67%) in the HID group (*p* < 0.001). In the MSD group, 5, 11, 6, and 3 patients developed grade I, grade II, grade III, and grade IV aGvHD, respectively, while in the HID group, 27, 21, 9, and 2 patients developed grade I, grade II, grade III, and grade IV aGvHD, respectively. As shown in **Figure 1**, the cumulative incidences of grade II–IV aGvHD at day 100 (*p* = 0.001) and mild to severe cGvHD at 5 years (*p* = 0.007) were significantly lower following MSD grafts vs. HID grafts. However, the cumulative incidences of grade III–IV aGvHD at day 100 (*p* = 0.237) and that of moderate to severe cGvHD at 5 years were similar (*p* = 0.076). MSD patients had a lower incidence of CMV (26% vs. 58%, *p* < 0.001) and EBV viremia (2% vs. 21%, *p* < 0.001) compared with HID patients. However, incidences of bloodstream infection (BSI) before neutrophil engraftment between the two groups were similar (*p* = 0.668).

By 28 days post-HSCT, 2 and 4 patients died in the MSD and HID groups, respectively. All 6 patients had infections prior to transplantation. In detail, 1 patient had mucormycosis confirmed with a bronchoscopy pre-HSCT and had a perianal swab before transplantation that grew carbapenem-resistant enterobacteriaceae (CRE); 1 patient had *Pseudomonas aeruginosa* (PA)-BSI at diagnosis, a nasal swab grew carbapenem-resistant *Klebsiella pneumoniae* (CRKP) colonization before transplantation, and nasopharyngeal and perianal swabs screened positive for CRPA and CRE colonization during conditioning; 1 patient had a disseminated infection of their lung and spleen, which was defined as SD and CRKP colonization pre-HSCT; 1 patient had CRPA-BSI at diagnosis and possibly an invasive pulmonary fungal disease defined as SD at transplantation; and 2 patients had prior long-term courses of broad-spectrum antibiotics pre-HSCT due to multiple sites of infection in partial remission at the time of transplantation. Five out of the 6 patients died of multidrug-resistant organism (MDRO)-BSI (n = 4) or progressive pulmonary infection (n = 1). GF occurred in 7 patients, of whom 3 received a secondary transplantation with 1 expired due to aGvHD 2 patients recovered with partial autologous blood recovery, 1 patient experienced blood recovery from a frozen donor stem cell infusion and supportive care, and 1 patient died from infection. With a median follow-up of 37 months (range, 4–87 months) and 25 months (range, 4–87 months), 14 and 23 patients died in the MSD and HID groups, respectively. As shown in **Table 2**, the leading causes of death were aGvHD (n = 8 vs. n = 9) followed by infection (n = 5 vs. n = 8) in the MSD and HID groups, respectively.

**Table 2:** Primary causes of death (COD) among patients who received allografts.

COD	MSD group (14)	HID group (23)
aGvHD	8	9
Infection	5	8
cGvHD	–	3
Graft failure	–	2
Suicide	1	–
Intracranial hemorrhage	–	1

MSD, matched sibling donor; HID, haploidentical donor; BFAC, conditioning regimen consisting of busulfan/fludarabine/antithymocyte globulin/cyclophosphamide; FAC, conditioning regimen consisting of fludarabine/antithymocyte globulin/cyclophosphamide; aGvHD, acute graft versus host disease; cGvHD, chronic graft versus host disease.

### IST Outcomes

Three months after IST, 6 patients were not evaluable due to death. In total, 182 of 188 patients were contacted. Of these, only 9 (5%) were in CR and 75 (41%) patients were in PR. A total of 173 patients were evaluable at 6 months, of whom 26 (15%) and 79 (46%) achieved CR and PR, respectively. One year after IST, a total of 18 were not evaluated for a response due to death (n = 17) or transplantation (n = 1). Of the 140 patients accessed, 43 (31%) were in CR and 60 (43%) were in PR. At the last follow-up on April 30, 2021, of the 157 evaluable patients, 79 (50%) were in CR and 46 (29%) were in PR. Compared with transplantation, IST had a significantly lower rate of complete blood count recovery at these timepoints (**Table 1**, *p* < 0.001). Nine patients underwent HID-HSCT due to treatment failure, 7 patients after non-response at 6 months, 1 patient with acute myelocytic leukemia (AML) transformation at 11 months, and 1 patient with MDS transformation at 12 months after initial IST and received HID-HSCT.

With a median follow-up of 23 months (range, 3–87 months), a total of 31 patients died. The causes of death included infection in 22 patients, gastrointestinal hemorrhage in 1 patient, intracranial hemorrhage in 6 patients, and MDS/AML evolution in 2 patients.

### Survival, TRM, and Clone Evolution

**Figure 2:**
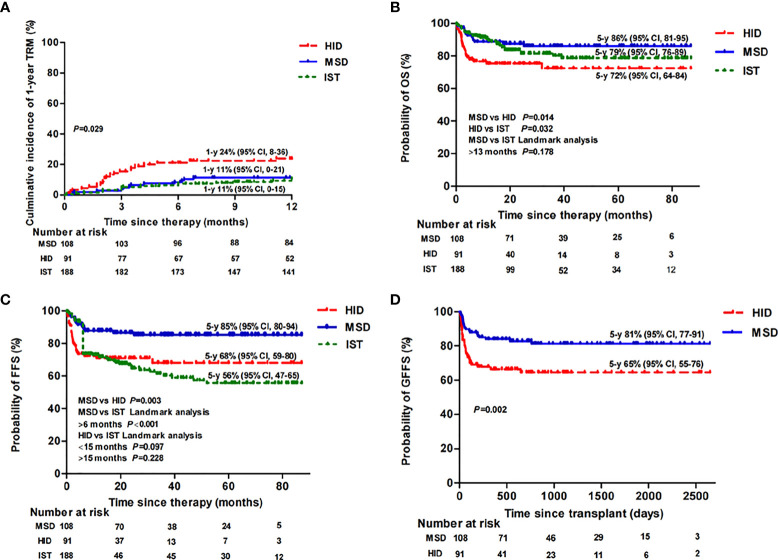
Therapy-related mortality (TRM) **(A)**, overall survival (OS) **(B)**, failure-free survival (FFS) **(C)**, and GvHD-free, failure-free survival (GFFS) **(D)** of MSD-HSCT, HID-HSCT, and IST patients.

The estimated 5-year OS rates were 86% [95% confidence interval (CI), 81–95], 72% (95% CI, 64–84), and 79% (95% CI, 76–89) (*p* = 0.02) (**Figure 2B**) among patients in the MSD, HID, and IST groups, respectively; accordingly, the estimated 5-year FFS rates were 85% (95% CI, 80–94), 68% (95% CI, 59–80), and 56% (95% CI, 47–65), respectively (*p* < 0.001) (**Figure 2C**). Both MSD-HSCT and IST had statistically superior OS rates over HID-HSCT (*p* = 0.014; *p* = 0.032). However, there was no difference in OS rate between the MSD and IST groups at a landmark point of 13 months after treatment (*p* = 0.178). There was a significant difference in FFS between the MSD and HID groups (*p* = 0.003) and the MSD and IST groups (*p* < 0.001). Landmark analysis was performed to compare the FFS of HID and IST patients. The differences were not statistically significant both before (*p* = 0.097) and after (*p* = 0.228) the landmark point of 15 months following therapy (**Figure 2C**). The estimated 5-year GFFS rate of MSD patients was 81% (95% CI, 77–91) vs. 65% (95% CI, 55–76) of HID patients (*p* = 0.002) (**Figure 2D**).

**Figure 3:**
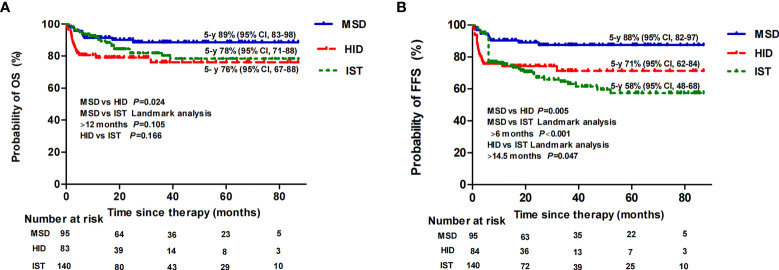
Overall survival (OS) **(A)** and failure-free survival (FFS) **(B)** following MSD-HSCT, HID-HSCT, and IST for patients aged ≤40 years.

In the subgroup analysis, when comparing patients aged ≤20 years to 20–40 years, the estimated 5-year OS rate of the MSD group was 95% (95% CI, 91–100) vs. 84% (95% CI, 76–98) (*p* = 0.144); the estimated 5-year OS rate of the HID group was 79% (95% CI, 70–94) vs. 79% (95% CI, 69–97) (*p* = 0.879); and the estimated 5-year OS rate of the IST group was 85% (95% CI, 77–99) vs. 74% (95% CI, 64–98) (*p* = 0.144), respectively. Thereafter, for patients aged ≤40 years, the estimated 5-year OS rate was 89% (95% CI, 83–98) following an MSD graft, which was statistically higher than following an HID graft (76% [95% CI, 67–88], p = 0.024), but not statistically higher than following IST when landmark analysis at 12 months was performed (78% [95% CI, 71–88], p =0.105). There was also no difference in OS between the HID and IST groups (p = 0.166) (**Figure 3A**). However, the estimated 5-year FFS rate was 88% (95% CI, 82–97) following an MSD graft (p = 0.001), which was significantly higher than following an HID graft (71% [95% CI, 62–84], p = 0.005) and IST (58% [95% CI, 48–68], p < 0.001) (**Figure 3B**). We performed a landmark analysis to compare the FFS before and after a landmark point of 14.5 months of HID and IST patients. HID patients had superior FFS compared with IST patients after that point (*p* = 0.047). For patients aged >40 years, owing to the small sample sizes of MSD (n = 13) and HID (n = 8) patients, we did not perform any further statistical analysis. The 5-year OS rate was 81% (95% CI, 72–97) among the IST older patients, which was equivalent to that of patients aged ≤20 or 20–40 years (*p* = 0.977).

The cumulative incidence of TRM 1 year after MSD, HID, and IST was 11% (95% CI, 0–21), 24% (95% CI, 8–36), and 11% (95% CI, 0–15) (*p* = 0.029), respectively (**Figure 2A**). Eleven patients (6%) in the IST group experienced MDS/AML evolution compared with none of patients in the transplant groups (*p* = 0.002). As expected, during follow-up, we noticed that young women (≤40 years) receiving a FAC conditioning regimen were more likely to restore menstruation after transplantation (9 out of 10) compared with those receiving a BFAC conditioning regimen (3 out of 10 patients) (*p* = 0.02).

### Multivariate Analysis

**Figure 4:**
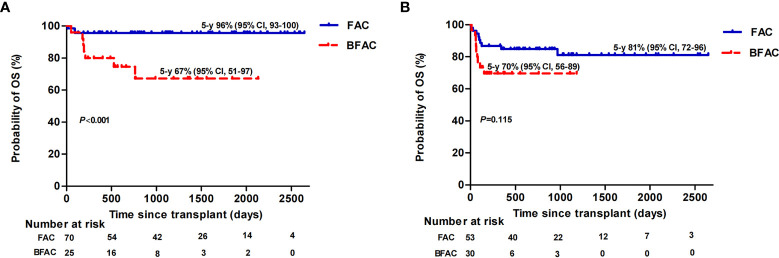
Overall survival (OS) of patients aged ≤40 years stratified by conditioning regimen in MSD **(A)** and HID **(B)** patients.

We performed univariate and multivariate analyses to identify risk factors associated with allograft survival (**Supplementary Table 2**). HID and a BFAC conditioning regimen were adverse factors for OS while HID and MDRO colonization were the only risk factors for FFS and GFFS, respectively. For patients aged ≤40 years undergoing transplantation, the P value of HID for OS in multivariate analyses was 0.05 with marginal significance, a BFAC conditioning regimen was the only risk factor for OS and FFS, and no risk factor for GFFS was identified. However, the addition of BU had an adverse effect on young MSD patients but not on young HID patients. In detail, the estimated OS at 5 years of young MSD patients receiving a BFAC conditioning regimen was 67% (95% CI, 51–97) vs. 96% for those receiving a FAC conditioning regimen (95% CI, 93–100) (*p* < 0.001), (**Figure 4A**), while the estimated OS rate at 5 years among young HID patients receiving a BFAC conditioning regimen was 70% (95% CI, 56–89) vs. 81% (95% CI, 72–96) for those receiving a FAC conditioning regimen (*p* = 0.115), (**Figure 4B**).

### Propensity Score Matching

We also performed propensity score matching to minimize effects of confounding donor factors (56 patients in the MSD and HID groups, respectively). The matched patient and donor baseline characteristics were not significantly different between the MSD and HID groups (*p* < 0.05) (**Supplementary Table 3**). The standardized mean differences were less than 0.2 (**Supplementary Image 1**). After matching, MSD-HSCT still exhibited significant superiority over HID-HSCT in terms of survival. In detail, the estimated 5-year OS, FFS, and GFFS rates of MSD patients were 92% (95% CI, 88–100), 92% (95% CI, 88–100), and 87% (95% CI, 80–100) vs. 77% (95% CI, 67–90) (*p* = 0.012), 75% (95% CI, 65–88) (*p* = 0.006), and 69% (95% CI, 58–84) (*p* = 0.026) of HID patients (**Supplementary Image 2**), respectively. However, none of the evaluated factors was recognized as an independent risk factor of OS in multivariate analysis.

## Discussion and Conclusion

SAA is usually a life-threatening disease with a high mortality rate due to bleeding and infections. An effective therapy is therefore critical. For candidates with SAA, MSD-HSCT is the first-line therapy, IST is the recommended choice for those without an MSD, and HSCT from an alternative donor could be considered if IST fails (6). However, treatment failures after IST are high, especially for long-term survivors (19). Of 386 children with SAA, even though a 10-year OS rate of 88% was achieved, FFS rate was only 56% (20). As finding a matched unrelated donor is time-consuming and has limitations, HID-HSCT emerged mostly as a salvage after IST failures but has shown to be equivalent to MSD (3) and has a superior FFS to IST (4, 5) as a first-line strategy in China. The present work was the first to our knowledge that compared these three procedures together, concluding similar as well as different findings compared to prior studies.

Patient age is still an independent negative factor for survival following HSCT. However, the survival gap between age groups may be narrowing, especially for those aged ≤40 years, which is ascribed to improvements in supportive care as well as the transplant procedure itself, such as modifications to the conditioning regimen and effective management of complications post-HSCT over the last decade. This is evidenced by the findings of a prior work on EBMT that reported superior survival of patients younger than 20 years (17). We did not observe differences in the survival of MSD-HSCT or HID-HSCT patients younger than 40 years (21).

Consistent with previous reports (20, 22, 23), our study showed that MSD-HSCT achieved superior outcomes in terms of OS and FFS compared with IST. Compared with MSD-HSCT, our study found that HID-HSCT had a significantly higher incidence of GF, TRM, aGvHD, viremia, cGvHD, and eventually a lower survival. Due to the heterogeneous effects of variables such as patient age on survival following HSCT and IST, we only performed univariate and multivariate analyses between the allografts. In multivariate analysis, HID-HSCT was also identified as an independent risk factor of OS and FFS. Xu et al. observed that the survival of HID-HSCT was compared to that of MSD-HSCT (3). This is different from our results. We observed that the HID-HSCT recipients showed a longer interval from diagnosis to HSCT, and more heavily red blood cell transfusions than the cohort reported by Xu et al., which may contribute to the relative inferior outcomes of our HID-HSCT recipients. In addition, we enrolled 8 (9%) patients who were older than 40 years in the HID-HSCT group. In those who were equal to and younger than 40 years, we observed that the OS rate was still significantly superior for MSD-HSCT receipients compared to HID-HSCT receipients (89% vs. 76%, *p* = 0.024). However, a prospective study could help to further identify the efficacy of our conditioning regimen in old patients. In addition, our study showed similar estimated 5-year OS among HID-HSCT patients in contrast to another study of China (72% vs. 74.8%) (24).

The benefits of transplantation declined with increasing age, especially among HID-HSCT patients (24). We therefore compared the survival following HID and IST in those patients younger than 40 years. There was no difference in OS between HID and IST patients younger than 40 years, whereas those after HID had a higher rate of FFS when follow-up was longer than 14.5 months and a rapid blood recovery compared with IST patients. These findings were consistent with reports from other centers in China (4, 5). In accordance with other studies (5, 20, 25–27), we noticed that more patients experienced MDS/AML evolution in the IST group than in the HSCT group (6% vs. 0, *p* = 0.002). This may ascribe to an advantage of HSCT over IST in eradicating potential clonal hematopoiesis at risk of evolving to AML/MDS, especially during long-term follow-up. Taking quality of life into consideration, cGvHD was the major concern of HID-HSCT compared to IST. However, in line with other studies in China (4, 5, 28), our work reported a culminative incidence of estimated 5-year moderate to severe cGvHD after HID-HSCT of 9% (95% CI, 0–18) vs. 3% (95% CI, 0–5) (*p* = 0.076) after MSD-HSCT, and no patients had severe cGvHD in the HID group.

Cardiac toxicity related to high-dose CY is of great concern to patients with SAA who are elderly or have poor cardiac function due to anemia with lethal cardiotoxicity up to 2.3% (29). The FAC regimen showed evidence of reduced toxicity and high engraftment rates (21, 30), but secondary GF was still as high as 12.7% with CY 100 mg/kg and R-ATG 10 mg/kg for MSD patients in another Korean study (21). As a result, in our work most (72%) MSD patients received a CY dose of 150 mg/kg plus a moderate dose of ATG (R-ATG 12.5 mg/kg or P-ATG 100–125 mg/kg), and BU was added to the regimens of those at a high risk of graft failures, such as patients who required heavy transfusions and had a long interval from diagnosis to transplantation and NSAA. Of note, only 1 patient was diagnosed with secondary GF in the MSD group. This was encouraging as we applied this reduced-intensity conditioning to SAA patients receiving MSD-HSCT independent of age without impairing engraftment but reducing TRM, which was similar to a previous report (30). However, it appeared that the addition of BU did not translate into a survival benefit for those MSD patients who had longer intervals from diagnosis to transplantation and required heavily additional RBC transfusions. As a comparison, another study of 26 patients who required heavy transfusions (median 54 units) and had long-time intervals (median 26 months) reported successful engraftment using a FAC regimen without a primary GF (31). Of note, we observed the fertility maintenance effect of a FAC over a BAFC conditioning regimen (*p* = 0.02).

In contrast, the effects of a BFAC conditioning regimen on HID-HSCT may be different from MSD-HSCT. With experience learned from alternative donors (32), HLA disparity is a major obstacle of GF of HID-HSCT. Another Chinese study reported that 3 out of 26 patients (12%) undergoing HID-HSCT had a graft failure based on the FAC conditioning regimen (33). Likewise, in our study there were 6 graft failures in the HID group but only 1 patient with a positive DSA received a BFAC conditioning regimen and OS was equivalent between the two conditioning regimens for younger patients. The rate of GF among HID patients in the present work was higher than reported by other studies based on BU and CY (200 mg/kg) (3, 5, 34). The addition of BU to a FAC conditioning regimen may reduce the rate of GF. However, TRM was still high mainly due to infections. A strategy to improve outcomes further may be *via* a reduced dose of CY, according to another Chinese study that reported the same conditioning regimen with a dose of CY 2,000 mg/m^2^ for enrolled patients (median age, 11 years) undergoing HID-HSCT. None of the enrolled patients had a primary or secondary GF in their study, and the 3-year OS rate was 80.3 ± 5.1% (35). Another widely used model of HID-HSCT is post-transplant cyclophosphamide (PTCY) for GvHD prophylaxis. However, the engraftment rates of PTCY may be relatively lower than methotrexate-containing regimens, although prospective data are not available (36, 37).

Our study had several limitations. First, it was a retrospective study with imbalanced basic characteristics between groups and choice of therapy based on clinical physician or patient preference. Although final conclusions therefore cannot be drawn from our study, our comparative data are from a single large transplant center over the same study period with relatively consistent protocols and showed similar survival rates compared with other studies. We also performed stratification, regression analysis, and propensity score matching to reduce these confounders, and similar conclusions were achieved. Second, few patients diagnosed with NSAA or SAA evolving from NSAA received a FAC conditioning regimen, making a sound comparison of conditioning regimens between these patients difficult. Third, data on the rates of full immune reconstitution after transplantation between different groups were unavailable. Fourth, the follow-up of our study was limited and the fertility maintenance of a FAC conditioning regimen needs to be confirmed in the future.

In conclusion, with respect to OS and FFS, our study showed that MSD is still the preferred first-line therapy for patients with SAA younger than 40 years. Meanwhile, for younger patients (≤40 years) without an MSD, HID-HSCT was a valid choice comparable with IST. A FAC conditioning regimen was feasible for MSD-HSCT, whereas the addition of busulfan to the FAC regimen was harmful to MSD patients but might be beneficial for HID patients. Follow-up prospective, large sample size multicenter studies are needed to identify the optimal alternative therapy and conditioning regimen for SAA patients.

## Data Availability Statement

The raw data supporting the conclusions of this article will be made available by the authors, without undue reservation.

## Ethics Statement

The studies involving human participants were reviewed and approved by the Ethics Committee of Institute of Hematology & Blood Diseases Hospital, Chinese Academy of Medical Sciences & Peking Union Medical College. Written informed consent to participate in this study was provided by the participants’ legal guardian/next of kin.

## Author Contributions

SF and YzZ contributed to study design and manuscript reviewing. YfZ and JH contributed to the data collection, analysis, and manuscript composition. LL, YS, JC, and TZ contributed to the data collection and interpretation. XC, AP, DY, RZ, QM, WZ, YH, JW, EJ, and MH contributed to the treatment of the disease and data collection. All authors contributed to the article and approved the submitted version.

## Funding

The author(s) disclosed receipt of the following financial support for the research, authorship, and/or publication of this article: This work was supported by the CAMS Innovation Fund for Medical Sciences (CIFMS) [grant numbers 2021-1-I2M-017 and 2021-I2M-C&T-B-080], Youth Program of National Natural Science Foundation of China (grant number 81900182), and the Non-profit Central Research Institute Fund of Chinese Academy of Medical Sciences [2019XK320076].

## Conflict of Interest

The authors declare that the research was conducted in the absence of any commercial or financial relationships that could be construed as a potential conflict of interest.

## Publisher’s Note

All claims expressed in this article are solely those of the authors and do not necessarily represent those of their affiliated organizations, or those of the publisher, the editors and the reviewers. Any product that may be evaluated in this article, or claim that may be made by its manufacturer, is not guaranteed or endorsed by the publisher.

## Supplementary Material

Supplementary Figure 1 Propensity score matching of patients undergoing transplantation.

Supplementary Figure 2 The estimated 5-year overall survival (OS) (A), failure-free survival (FFS) (B), and GVHD-free, failure-free survival (GFFS) (C) rates of MSD patients and HID patients after matching.

Supplementary Table 1 Characteristics and outcomes of patients with acquired aplastic anemia and their donors in the transplant groups.

Supplementary Table 2 Matched patient and donor baseline characteristics of MSD and HID patients.

Supplementary Table 3 Univariate analysis and multivariate analysis of all patients undergoing transplantation and only those aged≤40 years.
