# Supplementary figures and images for: Comparison of Hematopoietic Stem Cell Transplantation Outcomes Using Matched Sibling Donors, Haploidentical Donors, and Immunosuppressive Therapy for Patients With Acquired Aplastic Anemia
Source: Front Immunol. 2022 Feb 1;13:837335. doi: 10.3389/fimmu.2022.837335 (PMC8843935; doi:10.3389/fimmu.2022.837335)

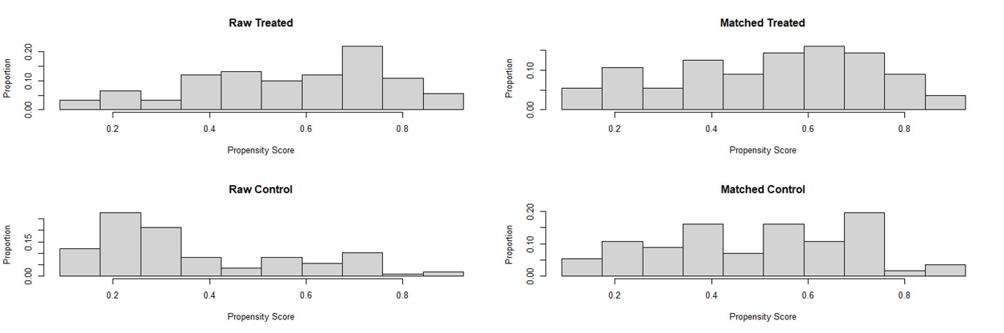

Supplement: Supplementary Figure 1 — Propensity score matching of patients undergoing transplantation. [file Image_1.tif]

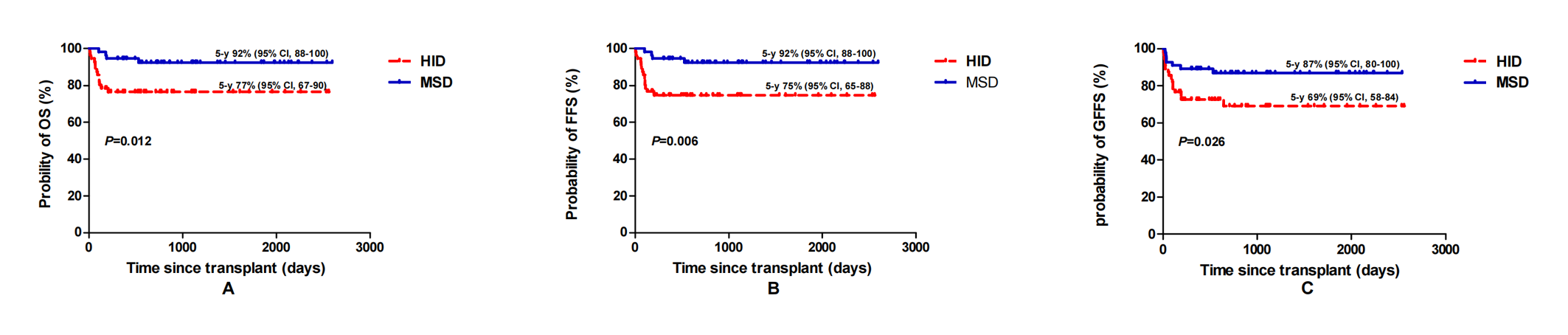

Supplement: Supplementary Figure 2 — The estimated 5-year overall survival (OS) (A), failure-free survival (FFS) (B), and GVHD-free, failure-free survival (GFFS) (C) rates of MSD patients and HID patients after matching. [file Image_2.tif]
