# Supplementary material for: Comparison of Hematopoietic Stem Cell Transplantation Outcomes Using Matched Sibling Donors, Haploidentical Donors, and Immunosuppressive Therapy for Patients With Acquired Aplastic Anemia
Source: Front Immunol. 2022 Feb 1;13:837335. doi: 10.3389/fimmu.2022.837335 (PMC8843935; doi:10.3389/fimmu.2022.837335)
Supplement: Supplementary Table 1 — Characteristics and outcomes of patients with acquired aplastic anemia and their donors in the transplant groups. [file Table_1.docx]

**Supplementary Table 1: Characteristics and outcomes of patients with acquired aplastic anemia and their donors in the transplant groups**

| **Variables** | **MSD group （108）** | **HID group**  **（91）** | ***P* value** |
| --- | --- | --- | --- |
| Donor sex (male), no. (%) | 51 (47) | 59 (65) | 0.019 |
| Donor age, years, median (range) | 26 (8-56) | 36 (8-62) | <0.001 |
| Donor age group, no. (%) |  |  | <0.001 |
| ≤20 | 41 (38) | 49 (54) |  |
| ＞20 ≤40 | 54 (50) | 34 (37) |  |
| ＞40 | 13 (12) | 8 (9) |  |
| Previous ATG therapy, no. (%) | 3 (3) | 13 (14) | 0.007 |
| Ferritin level before transplantation, ng/ml, median (range) | 1045 (29-6901) | 1229 (12-13095) | 0.134 |
| Units of red blood cell pre-HSCT, median (range) | 10 (0-82) | 12 (0-100) | 0.676 |
| Infections pre-HSCT, no. (%) |  |  | 0.009 |
| Complete remission | 4 (4) | 9 (10) |  |
| Partial remission and stable disease | 25 (25) | 8 (9) |  |
| MDRO colonization, no. (%) | 5 (5) | 7 (8) | 0.545 |
| HCT-CI≥2, no. (%) | 10 (9) | 4 (4) | 0.29 |
| ECOG≥3, no. (%) | 0 (0) | 3 (3) | 0.188 |
| Donor–recipient relationship, no. (%) |  |  | <0.001 |
| Mother–child | - | 17 (19) |  |
| Father–child | - | 38 (41) |  |
| Child–mother | - | 4 (4) |  |
| Child–father | - | 3 (3) |  |
| Siblings | 108 (100) | 30 (33) |  |
| Blood types of donors to recipients, no. (%) |  |  | 0.325 |
| Matched | 68 (63) | 47 (52) |  |
| Major mismatched | 18 (17) | 17 (19)  )) |  |
| Minor mismatched | 16 (15) | 17 (19) |  |
| Major and minor mismatched | 6 (6) | 10 (11) |  |
| HLA-matched, no. (%) |  |  | <0.001 |
| 5/10 | - | 41 (45) |  |
| 6/10 | - | 16 (17) |  |
| 7/10 | - | 10 (11) |  |
| 8/10 | - | 6 (7) |  |
| 9/10 | - | 5 (6) |  |
| 10/10 | 108 (100) | 13 (14) |  |
| Conditioning regimen, no. (%) |  |  | 0.186 |
| FAC | 76 (70) | 55 (60) |  |
| BFAC | 32 (30) | 36 (40) |  |
| Cyclophosphamide dose, no. (%) |  |  | 0.365 |
| 150mg/kg | 78 (72) | 71 (78) |  |
| 120mg/kg | 21 (19) | 11 (12) |  |
| 80-100mg/kg | 9 (8) | 9 (10) |  |
| **Supplementary Table 1 (Continued)** |  |  |  |
| **Variables** | **MSD group （108）** | **HID group**  **（91）** | ***P* value** |
| ATG source, no. (%) |  |  | <0.001 |
| Porcine | 73 (68) | 28 (31) |  |
| Rabbit | 35 (32) | 63 (69) |  |
| Graft source, no. (%) |  |  | 0.037 |
| Peripheral blood | 97 (90) | 71 (77) |  |
| Bone marrow±peripheral blood | 11 (10) | 20 (22) |  |
| Mononuclear cells, ×10^8^/kg, median (range) | 8.3 (3.1-25.0) | 10 (5.1-25.5) | 0.009 |
| CD34^+^cells, ×10^6^/kg, median (range) | 2.6 (1.5-6.6) | 3.4 (1.6-8.6) | <0.001 |
| CD3^+^cells, ×10^6^/kg, median (range) | 125.5 (6.7-384.1) | 145.9 (46.2-1726.5) | 0.002 |
| CD4^+^cells, ×10^6^/kg, median (range) | 61.0 (1.8-226.8) | 76.9 (20.2-377.5) | 0.001 |
| CD8^+^cells, ×10^6^/kg, median (range) | 47.1 (4.1-141.8) | 55.1 (17.3-259.4) | 0.013 |
| Bloodstream infection, no. (%) | 20 (19) | 20 (22) | 0.668 |
| Neutrophil engraftment, days, median (range) | 12 (8-19) | 12 (10-23) | 0.039 |
| Platelet engraftment, days, median (range) | 13 (7-37) | 14 (8-95) | 0.023 |
| 28-day neutrophil engraftment, no. (%) | 106 (100) | 88 (100) | 1 |
| 28-day platelet engraftment, no. (%) | 101 (94) | 67 (76) | <0.001 |
| Graft failure, no. (%) ^*^ | 1 (1) | 6 (7) | 0.062 |
| Primary graft rejection | 0 (0) | 2 (2) |  |
| Secondary graft rejection | 1 (1) | 4 (5) |  |
| Cytomegalovirus viremia, no. (%) | 28 (26)  ) | 53 (58) | <0.001 |
| Epstein–Barr virus viremia, no. (%) | 2 (2)  ) | 19 (21) | <0.001 |
| 100-day aGvHD grade II-IV, no. (%) * | 17 (16) | 30 (34) | 0.006 |
| 100-day aGvHD grade III-IV, no. (%) * | 8 (8) | 10 (11) | 0.507 |
| Moderate to severe cGvHD, no. (%) ^#^ | 2 (2) | 5 (7) | 0.133 |
| EBV-associated PTLD, no. (%) | 1 (1) | 3 (3) | 0.334 |
| Overall death, no. (%) | 14 (13) | 23 (25) | 0.041 |

* Patients who survived ≥28 days; # patients who survived ≥100 days

Abbreviations: MSD matched sibling donor, HID haploidentical donor, no. number of patients, ATG antithymocyte globulin, HSCT hematopoietic stem cell transplantation, MDRO multi-drug resistant organism, HCT-CI HCT comorbidity index, ECOG eastern cooperation oncology group, FAC conditioning regimen consisting of fludarabine/antithymocyte globulin/cyclophosphamide, BFAC conditioning regimen consisting of bulsulfan/fludarabine/antithymocyte globulin/cyclophosphamide, aGvHD acute graft versus host disease, cGvHD chronic graft versus host disease, PTLD post-transplant lymphoproliferative disorder
