# Supplementary material for: Comparison of Hematopoietic Stem Cell Transplantation Outcomes Using Matched Sibling Donors, Haploidentical Donors, and Immunosuppressive Therapy for Patients With Acquired Aplastic Anemia
Source: Front Immunol. 2022 Feb 1;13:837335. doi: 10.3389/fimmu.2022.837335 (PMC8843935; doi:10.3389/fimmu.2022.837335)
Supplement: Supplementary file 6 [file Table_4.docx]

**Supplementary Image 1** Propensity score matching of patients undergoing transplantation

**Supplementary Image 2** The estimated 5-year overall survival (OS) **(A)**, failure-free survival (FFS) **(B)**, and GVHD-free, failure-free survival (GFFS) **(C)** rates of MSD patients and HID patients after matching

**Supplementary Table 1** Characteristics and outcomes of patients with acquired aplastic anemia and donors in the transplant groups

**Supplementary Table 2** Univariate analysis and multivariate analysis of all patients undergoing transplantation and only those aged≤40 years

**Supplementary Table 3** Matched patient and donor baseline characteristics of MSD and HID patients
